# Predicting adherence to acupuncture appointments for low back pain: a prospective observational study

**DOI:** 10.1186/s12906-016-1499-9

**Published:** 2017-01-03

**Authors:** Felicity L. Bishop, Lucy Yardley, Cyrus Cooper, Paul Little, George Lewith

**Affiliations:** 1 Psychology, Faculty of Social and Human Sciences, University of Southampton, Building 44 Highfield Campus, Southampton, SO17 1BJ UK; 2 MRC Lifecourse Epidemiology Unit, University of Southampton, Southampton General Hospital, Tremona Road, Southampton, SO16 6YD UK; 3 Primary Care and Population Sciences, Aldermoor Health Centre, University of Southampton, Southampton, SO16 5ST UK

**Keywords:** Acupuncture, Adherence, Back pain, Health knowledge, Attitudes, Practice, Illness perceptions, Treatment beliefs

## Abstract

**Background:**

Acupuncture is a popular form of complementary and alternative medicine (CAM), but it is not clear why patients do (or do not) follow acupuncturists’ treatment recommendations. This study aimed to investigate theoretically-derived predictors of adherence to acupuncture.

**Methods:**

In a prospective study, adults receiving acupuncture for low back pain completed validated questionnaires at baseline, 2 weeks, 3 months, and 6 months. Patients and acupuncturists reported attendance. Logistic regression tested whether illness perceptions, treatment beliefs, and treatment appraisals measured at 2 weeks predicted attendance at all recommended acupuncture appointments.

**Results:**

Three hundred twenty-four people participated (aged 18–89 years, M = 55.9, SD = 14.4; 70% female). 165 (51%) attended all recommended acupuncture appointments. Adherence was predicted by appraising acupuncture as credible, appraising the acupuncturist positively, appraising practicalities of treatment positively, and holding pro-acupuncture treatment beliefs. A multivariable logistic regression model including demographic, clinical, and psychological predictors, fit the data well (*χ* ^2^ (21) = 52.723, *p* < .001), explained 20% of the variance, and correctly classified 65.4% of participants as adherent/non-adherent.

**Conclusions:**

The results partially support the dynamic extended common-sense model for CAM use. As hypothesised, attending all recommended acupuncture appointments was predicted by illness perceptions, treatment beliefs, and treatment appraisals. However, experiencing early changes in symptoms did not predict attendance. Acupuncturists could make small changes to consultations and service organisation to encourage attendance at recommended appointments and thus potentially improve patient outcomes.

## Background

The inclusion of acupuncture in clinical practice guidelines for chronic back pain encourages increased integration of acupuncture into mainstream healthcare systems [1, 2]. As more acupuncture is funded by and accessed through public health care systems, acupuncture research needs to expand beyond questions of efficacy and incorporate a focus on questions related to health services and healthcare delivery. Poor attendance at appointments contributes to wasted resources throughout health care [3, 4] and could reduce the effectiveness of acupuncture. This study therefore investigated the predictors of full attendance at recommended acupuncture treatments in a cohort of patients with low back pain (LBP), a common reason for using acupuncture [5, 6].

Attendance for a course of treatments can be conceptualised as a form of adherence – the extent to which patients follow specific recommendations that have been agreed with a health care practitioner [7]. Research on acupuncture has rarely focused explicitly on adherence. However, good adherence predicts better outcomes in other therapies, including among patients taking placebos [8]. This suggests that good adherence might also predict better outcomes in acupuncture. One research letter reported that only 13 of 32 participants in a small clinical study completed all ten acupuncture treatments, suggesting that attendance can be poor [9]. Major trials of individualised acupuncture for back pain have reported the number of appointments attended but not compared this to recommendations, making it difficult to ascertain adherence [10–12]. Qualitative studies suggest that after initiating treatment, patients particularly value aspects of the relationship with the acupuncturist (e.g. individualised holistic caring consultations; egalitarian or collaborative relationships; length of time with the acupuncturist; seeing the same acupuncturist) and immediate and longer-term health benefits (e.g. the treatment itself being relaxing and enjoyable; improvements in symptoms, wellbeing, and function; gaining new insights into one’s health and/or treatment) [13–18]. In one study patients valued more mundane practicalities of treatment (e.g. clinics running to time) [19]. Patients’ reasons for stopping acupuncture can include financial considerations (although some patients make sacrifices elsewhere to enable on-going access) and perceived lack of effect [13, 18]. In the NHS in particular, patients are dissatisfied with inflexible appointment times and fixed (short) courses of treatment [13, 16, 20] which could lead to poor attendance.

Research on adherence to other forms of complementary and alternative medicine (CAM) suggests that patients evaluate CAMs against multiple criteria including: congruence with health-related beliefs; impact on and insight into symptoms, wellbeing, and energy levels; the quality of the therapeutic relationship; and practicalities such as financial cost [21–25]. Quantitative studies suggest that continued or committed use of CAMs might be higher in people with greater on-going medical need [26] health worries [27] and pro-CAM attitudes such as holistic models of health [28]. In surveys, respondents describe stopping CAM because it is too expensive, has not had the desired effects, or has been completely effective [29, 30]. Different personality traits predict adherence to CAM in different studies, including absorption [31], agreeableness [32], and low neuroticism [33]. Dissatisfaction with biomedicine can trigger initial CAM use but appears to be less relevant to decisions about ongoing CAM use [21, 26, 28].

An extended Common-Sense Model (CSM) provides a comprehensive and testable framework within which to study adherence to treatment [34–36]. According to this model, patients hold abstract beliefs about illness (‘illness perceptions’) and treatments (‘treatment beliefs’) which inform decisions to initiate treatment. Studies confirm that illness perceptions and treatment beliefs are indeed associated with CAM use [37–39]. Having initiated treatment, patients then continue or discontinue it based on a combination of abstract beliefs and concrete experiences such as improvements in symptoms or side-effects [40–42]. The CSM further specifies that relationships between concrete experiences and abstract beliefs are bidirectional [43]. One implication of this is that experiencing early improvements during treatment should not only predict adherence but should also strengthen the illness perceptions and treatment beliefs that originally led to treatment uptake. Adherence research within this framework has focused mainly on illness perceptions and treatment beliefs in long-term medication regimes for chronic illness and has shown that illness perceptions are only weakly associated with adherence [44] while treatment beliefs are stronger, more proximal determinants of adherence [34, 45]. Patients with chronic illness are more adherent to prescribed medication when beliefs about personal need for the medicine outweigh any concerns about it [46].

**Fig. 1:**
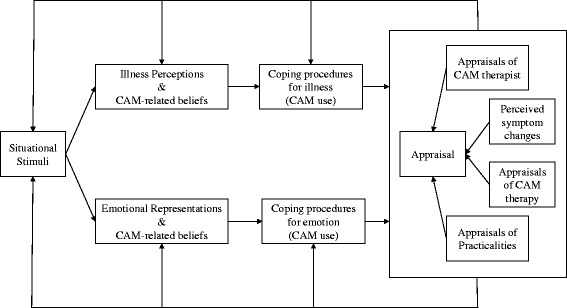
A dynamic extended common-sense model for CAM use. Adapted from [28] and originally adapted from the dynamic model of treatment perceptions [42] and the extended common sense model of self-regulation [34, 36]

We have previously adapted the extended CSM to study adherence to CAM by suggesting that patients appraise four particular aspects of CAM therapies: the therapy itself, the therapist, practicalities (e.g. convenience, cost), and symptom improvements early in treatment [28]. Qualitative data suggest relationships between these factors; for example, lack of improvement early in treatment might not deter patients if they are encouraged to continue treatment by their therapist [21, 42]. Appraisals of the therapy itself may be less important for adherence than appraisals of other aspects: in a longitudinal study adherence to CAM was predicted by positive appraisals of the therapist and practicalities but not the therapy [28]. More trusting and patient-centered therapeutic relationships have also been shown to increase adherence to biomedical treatments [47–49]. The dynamic extended CSM for CAM is shown in Fig. 1.

This study used the dynamic extended CSM for CAM to investigate adherence to acupuncture. The aim was to identify the predictors of full attendance at recommended acupuncture treatments in a cohort of patients with LBP. Specifically, we hypothesised that full attendance would be predicted by appraising the acupuncturist positively, appraising practicalities of treatment positively, experiencing early improvements in symptoms, holding pro-acupuncture treatment beliefs, and having positive perceptions of one’s LBP. We also hypothesised that experiencing early improvements in symptoms and appraising the acupuncturist positively would predict pro-acupuncture treatment beliefs and positive perceptions of LBP.

## Methods

### Design

The data for this analysis are drawn from a larger prospective observational cohort of patients with LBP receiving acupuncture [50]. Participants completed paper-based questionnaire measures of health and psychological variables four times – before starting treatment, 2 weeks, 3 months (when most courses of acupuncture for LBP have been completed), and 6 months later. Hypothesised predictors of adherence derived from the extended common-sense model were illness perceptions, treatment beliefs, and appraisals. This analysis used the 2-week measures of predictors unless otherwise specified (as pre-treatment beliefs should predict treatment initiation but not necessarily maintenance). The primary outcome was adherence at 3 months. The protocol was approved by Southampton and South West Hampshire Research Ethics Committee (A) (08/H0502/92) and data collection occurred between November 2008 and October 2010.

### Measures

All questionnaires were chosen for their theoretical relevance, psychometric properties, and brevity.

#### Predictors

Illness perceptions were measured using the validated and reliable 8-item Brief Illness Perceptions Questionnaire [51] worded specifically to assess perceptions of LBP. Eight single items assess perceptions of LBP as having severe consequences (consequences), lasting a long time (timeline), being controllable by the patient (personal control), being treatable (treatment control), causing many severe symptoms (identity), being worrying (concerns), being understandable (coherence), and having emotional effects (emotional). An open-ended question asks participants to identify three causes of their own LBP. All named causes were reviewed and inductively categorised, creating five separate binary variables. Fear avoidance beliefs and catastrophising can be conceptualised as illness perceptions specifically relevant to pain and were also included. Fear avoidance beliefs about physical activity and work were measured using the validated and reliable four and seven-item subscales from the Fear Avoidance Beliefs Questionnaire [52] (Cronbach’s α in this sample = 0.78 and 0.90 respectively). Catastrophising was measured using the validated and reliable 6-item subscale from the Coping Strategies Questionnaire [53] (α = 0.90).

Four dimensions of treatment beliefs were measured using the validated and reliable Complementary and Alternative Medicine Beliefs Inventory (CAMBI) [54] and Credibility Expectancy Questionnaire (CEQ) [55]. On the CAMBI, six items assessed holistic health beliefs (α = 0.67), six items assessed beliefs that treatments should be ‘natural’ or non-toxic (α = 0.83), and five items assessed belief that patients should actively participate in treatment (α = 0.63). On the CEQ, three items assessed expectations that acupuncture is an effective treatment for LBP (α = 0.94).

Four aspects of appraisals were measured. Appraisals of the acupuncturist were measured using the validated and reliable 10-item perceptions of therapist subscale from the Treatment Appraisal Questionnaire (TAQ) [28] (α = 0.91). Appraisals of the credibility of acupuncture for LBP were measured using the three item credibility subscale from the CEQ (α = 0.88). Appraisals of practicalities were measured using five single-items from the TAQ which were highly skewed and so negatively phrased items were reverse-scored and then all items dichotomised into “strongly agree” vs all other responses. Thus participants were classified according to whether they appraised their acupuncture as: value for money, not difficult to travel to, convenient appointments, not too much effort to attend, and not too expensive. Three dimensions of appraisals of early symptom changes were measured: back-related disability, using the 24-item Roland Morris Disability Questionnaire [56]; pain, using an 11-item numerical rating scale [57]; and wellbeing, using the single-item (100 mm visual analogue scale) Arizona Integrative Outcomes Scale [58]. Early symptom changes were assessed by calculating change scores on these three measures of health status, subtracting pre-treatment scores from 2-week scores. Continuous measures of health changes were used in the analyses of relationships among appraisals, illness perceptions and treatment beliefs, because linear relationships were hypothesised among these variables. For the analyses of predictors of adherence, participants were classified into five groups on each of the change variables (moderate improvement, small improvement, no change, small deterioration, moderate deterioration – see Table 1 for cut-offs). This classification facilitated investigation of non-linear relationships between health changes and adherence, e.g. any deterioration might discourage attendance, small improvements might encourage attendance, while large improvements might lead to early discontinuation.

**Table 1:** Cut points for health change categories

Category	Change scores in category		
	Disability (RMDQ)^a^	Pain (NRS)^b^	Wellbeing (AIOS)^c^
Small deterioration	1 to 4	1	−7 to 2
Moderate deterioration	> = 4	> = 2	<= − 7
No change	0	0	2–11
Small improvement	−1 to −4	−1	11 to 24
Moderate improvement	<= − 4	<= − 2	> = 24

Notes. ^a^ 4-point change on Roland Morris Disability Questionnaire is clinically/statistically meaningful [75]. ^b^ Patients view 2 point reduction on Numerical Rating Scale as moderately meaningful [76]. ^C^ No guidance available for Arizona Integrative Outcome Scale so quintile split used

#### Outcome

The duration of acupuncture treatment is often evaluated on an on-going basis and negotiated between patient and acupuncturist, resulting in individualised recommendations for the number of treatments (although this is less common in the NHS than the private sector [59]). Adherence was therefore operationalised as the extent to which patients attended all appointments as recommended by/agreed with their acupuncturist. Participants reported this on a 7-point likert scale. Acupuncturists reported, for each patient, the number of appointments recommended and the number attended. Acupuncturists’ recommendations were made based on usual clinical practice. A dichotomous measure of adherence (complete attendance vs incomplete attendance) was computed based on a combination of acupuncturists’ and participants’ reports – for a patient to be categorised as adherent both the patient and their acupuncturist had to report complete attendance.

### Procedure and participants

Eighty three acupuncturists (24 male, 59 female) were recruited from CAM clinics, general practice, pain clinics, and physiotherapy departments across Great Britain and Northern Ireland. They distributed baseline questionnaire packs (including information leaflets and consent forms) to consecutive eligible patients (aged over 18, scoring at least four on the Roland Morris Disability Questionnaire [56], no malignant pain). Patients returned questionnaires and consent forms to the researchers in Freepost envelopes. Subsequent questionnaires were mailed directly to patients. Gift vouchers and personalised and repeated follow-ups were used to enhance recruitment and retention [60, 61]. Four hundred and eighty five patients were recruited, of whom 324 provided attendance data and were included in this analysis.

### Statistical analyses

Missing values analysis in SPSS showed the proportion of missing data was low and Missing at Random, thus missing values were imputed using EM [62]. Analysis in MLWin confirmed that there was no significant effect on adherence of clustering of patients within clinics and so no adjustments for clustering were required.

To test hypotheses concerning predictors of adherence, SPSS was used to compute a series of univariable logistic regressions. Significant predictors (at *p* < .10) were entered into multivariable hierarchical logistic regression to identify independent predictors of adherence and to assess whether psychological variables predict adherence after controlling for demographic and clinical characteristics. Clinical and demographic variables were entered in Block 1, psychological variables were entered in Block 2. Variables were forced to enter the model within each block. The appropriateness of the data for logistic regression was tested. The Box-Tidwell procedure confirmed the data satisfied the assumption of linearity of the logit [63]. A linear regression was run and collinearity diagnostics examined; this confirmed there was no multicollinearity: all tolerance values > 0.1, all VIF < 10, no evidence of dependence in the variance of the regression co-efficients [64]. To test hypothesises concerning relationships among treatment appraisals, treatment beliefs and illness perceptions, partial correlations were conducted between continuous measures of these variables (controlling for baseline treatment beliefs and illness perceptions).

## Results

### Demographic and clinical characteristics and adherence

Table 2 summarises participants’ characteristics and presents the results of univariable logistic regressions to predict adherence. A slight majority of participants (51%, *n* = 165) attended all their recommended appointments. Participants were aged between 18 and 89 years old (M = 55.9, SD = 14.4). The majority were female, had chronic LBP, had not had acupuncture before, were receiving acupuncture in the public sector and were having other treatment(s) alongside acupuncture. Patients who were more likely to attend all their acupuncture appointments were older, had previous experience of acupuncture, were receiving additional treatments, were receiving acupuncture in the NHS, and were receiving acupuncture in a physiotherapy, GP, or pain clinic. Characteristics that were not significantly associated with attendance were: gender, duration of LBP, having a comorbid condition, and economic factors (see Table 2).

**Table 2:** Descriptive statistics and simple logistic regression analyses of demographic and clinical characteristics predicting attendance (*n* = 324)

Characteristic	Descriptive statistics		Regression results			
	f	%	Odds Ratio	95% Confidence Interval		*p*
				Lower	Upper	
Personal characteristics						
Age	---	---	1.02*	1.00	1.04	.012
Gender						
Female	228	70.4	1.51	0.93	2.43	.094
Education						.743
Left school aged <16 years ^a^	38	11.7				
Educated to 16	136	42	1.04	0.51	2.14	.909
Educated to 18	80	24.7	0.90	0.42	1.95	.789
Post-school education	70	21.6	0.76	0.34	1.67	.492
Economic factors						
Compensation claim pending	30	9.3	0.96	0.45	2.04	.915
Receiving back-related benefits	58	17.9	1.34	0.76	2.38	.316
Employment						.259
Employed at usual work ^a^	109	33.6				
On restricted duties	72	22.2	1.12	0.61	2.03	.718
Unpaid work (house work, student, retired)	143	44.1	1.50	0.91	2.47	.114
Clinical factors						
Prior acupuncture	133	41	1.61*	1.03	2.52	.037
Comorbidity	156	48.1	1.32	0.85	2.04	.217
Co-treatment	256	79	1.78*	1.03	3.06	.039
LBP duration						.429
Acute (<6 weeks) ^a^	41	12.7				
Persistent (6–52 weeks)	103	31.8	0.72	0.35	1.50	.385
Chronic (>52 weeks)	180	55.6	0.99	0.50	1.95	.970
Clinic characteristics						
Sector						
Private	111	34.3	0.50*	0.31	0.80	.004
Acupuncture style						.059
Unclear ^a^	60	18.5				
Western	164	50.6	0.97	0.54	1.76	.922
Traditional or TCM	85	26.2	0.52	0.27	1.01	.055
Mixed	15	4.6	1.64	0.50	5.37	.416
Clinic type						.070
CAM or acupuncture/TCM ^a^	96	29.6				
Physiotherapy	83	25.6	1.90*	1.05	3.44	.035
Pain clinic	95	29.3	1.85*	1.04	3.28	.037
GP	50	15.4	2.11*	1.05	4.22	.035

Notes. *p < .05. **p < .01. ^a^ Reference category

### Psychological variables and adherence

Table 3 summarises scores on the psychological variables and presents the results of univariable logistic regressions to predict adherence. One illness perception dimension predicted adherence: the odds of attending all appointments decreased with perceptions that one’s LBP causes many severe symptoms (illness identity). Two dimensions of treatment beliefs predicted adherence: the odds of attending all appointments increased with higher expectations of effectiveness and stronger preferences for participating in treatment. Five dimensions of appraisals predicted adherence: the odds of attending all appointments increased with more positive appraisals of the acupuncturist, appraisals of acupuncture as credible, and appraising appointments as convenient, not too much effort to attend, and affordable. Changes in disability, wellbeing, or pain scores in the first 2 weeks of treatment did not predict adherence.

**Table 3:** Descriptive statistics and univariable logistic regression analyses of adherence on psychological variables (*n* = 324)

	Descriptive statistics				Regression results			
	f	%	M	SD	Odds Ratio	95% C.I.		*p*
						Lower	Upper	
Illness perceptions								
LBP as threatening	---	---	47.63	10.73	0.99	0.97	1.01	.173
Consequences			6.82	2.11	1.00	0.91	1.11	.935
Timeline			7.78	2.18	0.97	0.88	1.07	.514
Personal control			4.54	2.33	1.03	0.94	1.13	.580
Treatment control			6.62	2.18	1.10	1.00	1.22	.058
Identity			6.95	1.86	0.89	0.79	1.00	.050
Concerns			7.86	1.96	0.99	0.88	1.10	.792
Comprehensible			6.83	2.57	1.08	0.99	1.18	.085
Emotional			6.21	2.46	1.01	0.93	1.11	.800
Caused by activities of daily living	120	37.0	---	---	0.89	0.57	1.40	.627
Caused by work	140	43.2	---	---	0.85	0.55	1.32	.460
Caused by accident/injury	123	38.0	---	---	0.83	0.53	1.29	.405
Caused by aging/genes	92	28.4	---	---	1.07	0.66	1.74	.777
Caused by disease	102	31.5	---	---	1.06	0.66	1.70	.801
Fear avoidance– physical activity			14.54	5.52	0.98	0.94	1.02	.301
Fear avoidance– work			15.17	13.11	0.97	0.98	1.01	.599
Catastrophising			2.45	1.43	0.93	0.80	1.08	.351
Treatment beliefs								
Expectancy	---	---	0.08	2.79	1.11*	1.02	1.21	.014
Holistic health	---	---	30.38	5.54	0.99	0.96	1.03	.785
Natural treatments	---	---	31.86	6.60	1.00	0.97	1.03	.988
Participation in treatment	---	---	26.85	4.88	1.05*	1.01	1.10	.030
Appraisals								
Credibility of acupuncture	---	---	0.11	2.64	1.08*	1.00	1.17	.046
Acupuncturist	---	---	60.31	8.86	1.03*	1.00	1.06	.028
Change in disability								.112
No change ^a^	49	15.1	---	---				
Small deterioration	78	24.1	---	---	1.28	0.63	2.62	.500
Moderate deterioration	16	4.9	---	---	2.29	0.69	7.58	.174
Small improvement	108	33.3	---	---	0.74	0.38	1.47	.393
Moderate improvement	73	22.5	---	---	1.41	0.68	2.92	.353
Change in pain								.777
No change ^a^	74	22.8	---	---				
Small deterioration	50	15.4	---	---	0.92	0.45	1.89	.827
Moderate deterioration	31	9.6	---	---	1.07	0.46	2.47	.880
Small improvement	75	23.1	---	---	1.34	0.70	2.56	.370
Moderate improvement	94	29.0	---	---	0.92	0.50	1.69	.784
Change in Wellbeing								
No change ^a^	67	20.7	---	---				.637
Small deterioration	64	19.8	---	---	1.50	0.75	2.99	.250
Moderate deterioration	69	21.3	---	---	1.00	0.51	1.97	.994
Small improvement	62	19.1	---	---	1.33	0.66	2.66	.422
Moderate improvement	62	19.1	---	---	0.96	0.48	1.92	.911
Value for money	92	28.4	---	---	1.55	0.95	2.52	.079
Not difficult to travel	184	56.8	---	---	1.12	0.72	1.74	.607
Convenient appointments	106	32.7	---	---	1.67*	1.04	2.67	.033
Not effortful to attend	219	67.6	---	---	2.29**	1.42	3.70	.001
Not too expensive	118	36.4	---	---	1.90**	1.20	3.01	.006

Notes. *p < .05. **p < .01. ^a^ Reference category

Table 4 presents the results of the hierarchical multivariable logistic regressions to predict adherence. Demographic and clinical characteristics were entered in Block 1 and this model was a good fit to the data (*χ* ^2^ (11) = 25.899, p = .007), but explained only approximately 10% of the variance in attendance (Nagelkerke’s R^2^ = 0.102), and resulted in 62.7% of participants being correctly classified as adherent/non-adherent. Adding psychological variables in Block 2 significantly improved model fit (*χ* ^2^ (10) = 26.824, p = .003). The final model including demographic, clinical, and psychological variables was a good fit to the data (*χ* ^2^ (21) = 52.723, *p* < .001), explained approximately 20% of the variance in attendance (Nagelkerke’s R^2^ = 0.200), and resulted in 65.4% of participants being correctly classified as adherent/non-adherent. Two variables remained as a significant independent predictor of adherence: the odds of attending all appointments were 1.23 times lower for every 1-unit increase in illness identity (perceptions that LBP causes many severe symptoms) and 2.09 times higher for participants who strongly disagreed that “seeing my therapist can be too much effort”.

**Table 4:** Multiple logistic regression analysis of predictors of attendance (*n* = 324)

	Odds ratio	95% C.I.		*p*
		Lower	Upper	
Demographic/Clinical characteristics				
Gender				
Female	1.40	0.81	2.41	.223
Age	1.01	0.99	1.03	.240
Prior acupuncture	1.55	0.93	2.59	.094
Co-treatment	1.46	0.78	2.76	.240
Sector				
Private	0.33	0.09	1.19	.089
Clinic type				
CAM or acupuncture/TCM ^a^				.820
Physiotherapy	0.93	0.24	3.64	.921
Pain clinic	0.74	0.16	3.56	.710
GP	0.63	0.13	3.11	.566
Acupuncture style				.660
Unclear ^a^				
Western	0.79	0.39	1.61	.520
Traditional or TCM	0.69	0.31	1.55	.368
Mixed	1.32	0.37	4.79	.671
Psychological variables				
Illness perceptions				
Treatment control	0.99	0.83	1.17	.869
Identity	0.81**	0.90	0.94	.006
Comprehensible	1.04	0.93	1.15	.521
Treatment beliefs				
Expectancy	1.10	0.94	1.28	.259
Participation in treatment	1.05	1.00	1.11	.072
Appraisals				
Credibility of acupuncture	0.96	0.81	1.14	.663
Acupuncturist	1.00	0.97	1.03	.850
Convenient appointments	1.40	0.79	2.45	.254
Not effortful to attend	2.09*	1.18	3.73	.012
Not too expensive	1.23	0.72	2.08	.454

Notes. *p < .05. **p < .01. ^a^ Reference category

### Appraisals, illness perceptions, and treatment beliefs

Table 5 summarises the partial correlations between appraisals and illness perceptions and treatment beliefs. After controlling for baseline beliefs, appraising acupuncture as credible, appraising the acupuncturist positively, and experiencing early health improvements were all associated with positive treatment beliefs and illness perceptions 2 weeks into treatment. In particular, positive appraisals were associated with higher expectations of acupuncture’s effectiveness, perceiving higher levels of personal and treatment-related control over LBP, and perceiving one has a good understanding of LBP. Out of the four dimensions of appraisals, experiencing early health improvements were the most strongly and consistently associated with treatment beliefs and illness perceptions 2 weeks into treatment. Appraisals of practicalities of treatment were only weakly associated with illness perceptions and treatment beliefs. Positive appraisals were not associated with more general CAM-related beliefs.

**Table 5:** Partial correlations between appraisals, illness perceptions and treatment beliefs

	Appraisals									
	Credibility of Acupuncture	Acupuncturist	Disability change	Pain change	Wellbeing change	Value for money	Not difficult to travel	Convenient appointments	Not effortful to attend	Not too expensive
Illness perceptions										
Consequences	-.10	-.06	.34**	.34**	-.29**	.04	.02	.05	.03	-.05
Timeline	-.16**	-.05	.25**	.26**	-.22**	-.05	-.02	-.02	.01	-.09
Personal control	.19**	.14*	-.23**	-.13*	.17**	.09	.10	.03	.11	.06
Treatment Control	.62**	.28**	-.20**	-.26**	.24**	.16**	.07	.16**	.11*	.06
Identity	-.10	.03	.34**	.30**	-.16**	.04	.01	.01	.06	-.12*
Concerns	-.14*	-.08	.26**	.26**	-.18**	-.03	-.02	-.06	-.10	-.12*
Comprehensible	.36**	.19**	-.17**	-.14*	.18**	.18**	.04	.07	.08	.04
Emotional	-.13*	-.01	.25**	.24**	-.15**	-.04	-.07	-.10	-.10	-.10
Treatment beliefs										
Expectancy	.70**	.28**	-.22**	-.33**	.27**	.16**	.11	.08	.08	.03
Holistic health	.06	.15**	-.02	-.04	.08	.05	-.05	.01	-.02	-.06
Natural treatments	.01	.11*	-.07	-.08	.06	-.01	-.03	.01	-.09	-.10
Participation in treatment	.03	.05	.02	.03	-.13*	.09	.07	.01	.07	.08

Notes. Each partial correlation controls for the baseline score on the relevant illness perception/treatment belief. *p < .05. **p < .01

## Discussion

Data from a longitudinal prospective cohort study were used to investigate the predictors of complete attendance for a course of acupuncture for LBP. As hypothesised, adherence to appointments was predicted by appraising acupuncture as credible, appraising the acupuncturist positively, appraising practicalities of treatment positively, and holding pro-acupuncture treatment beliefs. Contrary to predictions, experiencing early changes in symptoms did not predict attendance, which makes it likely that our findings are not conflated by treatment effects. Experiencing early symptom improvements and appraising acupuncture and the acupuncturist positively were all associated with higher expectations of acupuncture’s effectiveness and perceptions of LBP as more controllable and comprehensible.

In the univariable models, patients who had higher odds of attending all of their acupuncture appointments were older, had previous experience of acupuncture, were receiving additional treatments, were receiving acupuncture in the NHS, and were receiving acupuncture in physiotherapy, GP, or pain clinics (compared to acupuncture or CAM clinics). This suggests that previous acupuncture users are more committed to treatment than patients new to acupuncture, probably because the former are returning for a treatment they previously found effective. Receiving additional treatments might indicate worse overall health which could increase motivation for acupuncture. Acupuncture users in the NHS might be more likely than those in the private sector to adhere to appointments because they are grateful for free access to acupuncture [13] and/or because they are more likely to have shorter treatment courses of fixed duration [59]. Physiotherapy, GP and pain clinics are more likely to be in the NHS than are CAM clinics, which might explain increased adherence in the former settings. The impact of age was small - for each additional year of age the odds of attending all appointments were 1.02 times higher – and may indicate increasing commitment to health in general with increasing age. A recent large-scale study of adherence to medications in chronic illness found that older adults were also more likely to adhere to medications [65].

Odds of attending all acupuncture appointments increased with: weak perceptions that LBP causes many severe symptoms; high expectations of effectiveness and strong preferences for participating in treatment; and positive appraisals of the acupuncturist, appraisals of acupuncture as credible, and appraisals of acupuncture appointments as convenient, affordable, and not effortful to attend. Patients who do not associate lots of severe symptoms with their LBP might be more able physically to attend acupuncture appointments, which would lead to increased adherence (although LBP severity was not associated with adherence). Alternatively, patients who associate lots of severe symptoms with their LBP might feel they need a comprehensive multidisciplinary treatment to address these symptoms. While traditional acupuncture typically addresses the patient as a whole rather than focusing on a single symptom or condition, non-traditional acupuncture (e.g. Western styles) may be more symptom-focussed [66] and only approximately 25% of patients in this study were receiving traditional acupuncture. It is worth noting however that acupuncture style did not in itself predict adherence in this study.

People who expected acupuncture to be effective and believed it is important to participate in treatment were more likely to attend all their appointments, which can be understood as demonstrating the tendency towards common-sense coherence between treatment beliefs and adherence suggested by the extended common-sense model [34–36]. That participants apparently appraised multiple aspects of treatment when deciding whether to continue attending appointments is consistent with qualitative research in which patients evaluated multiple aspects of CAM when deciding whether to continue treatment [21]. This finding also supports the explication of multiple dimensions of appraisal in the dynamic extended common sense model for CAM [28]. Experiencing early changes in symptoms did not predict attendance, which can be explained if participants, like other CAM users [21], preferred to delay judging the effectiveness of therapy or if acupuncturists, like chiropractors [42] and rehabilitation therapists [67], reassured patients and helped them to interpret early changes (or lack thereof) positively.

In the final logistic regression model, a combination of demographic, clinical and psychological variables accounted for 20% of the variance in complete attendance. Psychological and other variables contributed similar amounts, confirming that attendance is dependent on multiple factors of different types. However, only two variables emerged as significant predictors of attendance (perceiving that one’s LBP causes many severe symptoms and perceiving acupuncture appointments as not too much effort to attend) which suggests shared variance among the predictors and possible mediation effects. A large proportion of variance in attendance (80%) remained unaccounted for by our predictors. This is broadly comparable to our previous study of adherence to diverse CAM therapies in which illness perceptions, treatment beliefs and treatment appraisals explained 25% of the variance in attendance, 19% of the variance in adherence to lifestyle recommendations and 39% of the variance in adherence to remedies [28]. These findings strongly suggest that additional variables to the illness perceptions, treatment beliefs, and treatment appraisals measured in this study are needed to understand and predict complete attendance for acupuncture and other CAM treatments. Such variables might include not only other beliefs, such as perceived need for and concerns about treatment [46, 68], health locus of control (previously associated with acupuncture use [69]), and health-related self-perceptions (previously predicted CAM use [70]), but also social constructs such as social network characteristics (associated with CAM use [71]) and social support (associated with adherence to biomedical therapies [72]). Alternative measures of some constructs might have been more appropriate. For example, self-rated health changes may be more strongly related to adherence than researcher-computed health changes; other measures related to the therapeutic relationship such as working alliance [73] might better capture the impact of therapist-patient communication on adherence [42].

Strengths of this study include its prospective design, use of reliable and previously validated measures of multiple predictors of adherence derived from an established theoretical framework, and the comparatively large sample of acupuncture patients drawn from diverse clinics across the UK. The lack of an objective measure of attendance is a limitation, although the possible bias introduced by self-report measures of adherence is somewhat mitigated by the combined use of patient and practitioner reports. It is possible that acupuncture patients who volunteer to participate in research are more likely to adhere to treatment than patients who do not volunteer, and if this were the case then this study may have overestimated adherence rates. We could locate no comparable published data on adherence in practice to test this possibility.

The results suggest several ways in which acupuncturists could encourage patients to attend all recommended appointments and thus probably improve patient outcomes. Patients who associated lots of severe symptoms with their LBP were less likely to adhere, so acupuncturists from all traditions could ensure they discuss and address diverse symptoms and comorbidities with patients. Patients who appraised their acupuncturist positively - finding them to be trustworthy and good communicators - were more likely to attend than other patients. This is consistent with previous studies in CAM [28] and biomedicine [74], and reinforces the importance of good communication and relationship-building skills for encouraging adherence to treatment recommendations. Acupuncturists may also be able to structure their services to facilitate adherence and minimise the practical barriers that were associated with incomplete attendance in this study. This would entail offering more convenient and affordable appointments that patients can easily fit into their lives and attend with minimal effort. Acupuncturists could consider asking patients to complete the five practical items from the TAQ [28] early in treatment to identify patients most at risk of early discontinuation and open up a discussion with them about ways to ease the burden of attending appointments.

## Conclusions

In conclusion, the results broadly supported the dynamic extended CSM for CAM use. Adherence to acupuncture was predicted by patients’ perceptions of their LBP, their expectations of acupuncture, and their appraisals of their early experiences of their acupuncturist, the credibility of acupuncture, and the practicalities of attending appointments. Contrary to predictions, experiencing early changes in symptoms did not predict attendance. We have suggested several ways in which acupuncturists could encourage patients to attend all recommended appointments. Future research should explore additional variables to improve our understanding of adherence to acupuncture.
